# Supplementary material for: AffyMAPSDetector: a software tool to characterize Affymetrix GeneChip™ expression arrays with respect to SNPs
Source: BMC Bioinformatics. 2007 Jul 30;8:276. doi: 10.1186/1471-2105-8-276 (PMC1959249; doi:10.1186/1471-2105-8-276)
Supplement: Additional file 13 — AffyMAPSDetector v1 source code. AffyMAPSDetector source code. [file 1471-2105-8-276-S13.zip › AffyMAPSDetector_v1_src/doc/affychip/snpdetector/resources/AppIcons.html]

AppIcons


|  |  |  |  |
| --- | --- | --- | --- |
| **Overview** \| **Package** \| **Class** \| **Use** \| **Tree** \| **Deprecated** \| **Index** \| **Help** | | |  |
| PREV CLASS   **NEXT CLASS** | **FRAMES**    **NO FRAMES**     **All Classes** |  |  |
| SUMMARY: NESTED \| FIELD \| CONSTR \| METHOD | DETAIL: FIELD \| CONSTR \| METHOD |  |  |


---


## affychip.snpdetector.resources Class AppIcons

```
java.lang.Object
  affychip.snpdetector.resources.AppIcons
```

---

public class **AppIcons** extends java.lang.Object

Title: Probe SNP Detector

Description: Image icons used by the UI components

Copyright: Sunita Kumari -- Copyright (c) 2004

Company: George Mason University

**Version:**
:   1.1

**Author:**
:   Sunita Kumari

---

|  |  |
| --- | --- |
| **Field Summary** | |
| `static javax.swing.ImageIcon` | `ICON_ABOUT` |
| `static javax.swing.ImageIcon` | `ICON_CLOSE` |
| `static javax.swing.ImageIcon` | `ICON_CONNECT` |
| `static javax.swing.ImageIcon` | `ICON_EXIT` |
| `static javax.swing.ImageIcon` | `ICON_HELP` |
| `static javax.swing.ImageIcon` | `ICON_LOGO` |
| `static javax.swing.ImageIcon` | `ICON_PLAY` |
| `static javax.swing.ImageIcon` | `ICON_SEARCH` |
| `static javax.swing.ImageIcon` | `ICON_STOP` |
| `static javax.swing.ImageIcon` | `ICON_WIZARD` |


**Constructor Summary**

`AppIcons()`


|  |  |
| --- | --- |
| **Method Summary** | |
| `protected static javax.swing.ImageIcon` | `createImageIcon(java.lang.String filename)` |

**Methods inherited from class java.lang.Object**

`clone, equals, finalize, getClass, hashCode, notify, notifyAll, toString, wait, wait, wait`

**Field Detail**

### ICON\_ABOUT

```
public static javax.swing.ImageIcon ICON_ABOUT
```

---


### ICON\_SEARCH

```
public static javax.swing.ImageIcon ICON_SEARCH
```

---


### ICON\_STOP

```
public static javax.swing.ImageIcon ICON_STOP
```

---


### ICON\_EXIT

```
public static javax.swing.ImageIcon ICON_EXIT
```

---


### ICON\_CLOSE

```
public static javax.swing.ImageIcon ICON_CLOSE
```

---


### ICON\_CONNECT

```
public static javax.swing.ImageIcon ICON_CONNECT
```

---


### ICON\_PLAY

```
public static javax.swing.ImageIcon ICON_PLAY
```

---


### ICON\_HELP

```
public static javax.swing.ImageIcon ICON_HELP
```

---


### ICON\_WIZARD

```
public static javax.swing.ImageIcon ICON_WIZARD
```

---


### ICON\_LOGO

```
public static javax.swing.ImageIcon ICON_LOGO
```


**Constructor Detail**

### AppIcons

```
public AppIcons()
```


**Method Detail**

### createImageIcon

```
protected static javax.swing.ImageIcon createImageIcon(java.lang.String filename)
```


---


|  |  |  |  |
| --- | --- | --- | --- |
| **Overview** \| **Package** \| **Class** \| **Use** \| **Tree** \| **Deprecated** \| **Index** \| **Help** | | |  |
| PREV CLASS   **NEXT CLASS** | **FRAMES**    **NO FRAMES**     **All Classes** |  |  |
| SUMMARY: NESTED \| FIELD \| CONSTR \| METHOD | DETAIL: FIELD \| CONSTR \| METHOD |  |  |


---
